# Supplementary material for: Liubao Tea Extract Attenuates High-Fat Diet and Streptozotocin-Induced Type 2 Diabetes in Mice by Remodeling Hepatic Metabolism and Gut Microbiota
Source: Nutrients. 2025 Aug 18;17(16):2665. doi: 10.3390/nu17162665 (PMC12389309; doi:10.3390/nu17162665)
Supplement: Supplementary file 1 [file nutrients-17-02665-s001.zip › nutrients-3765082-supplementary.pdf]

Supplementary methods

Preparation of Liubao tea extract (LBTE) from Liubao tea

The Liubao tea (LBT) was completely pulverized, and a 70% ethanol solution was added at a liquid-to-material ratio of 20:1. The mixture was heated in a water bath at 60 °C for 2 hours, with shaking every 30 minutes to ensure uniform mixing. After cooling, the solution was filtered. The filtrate was collected, and the residue was subjected to two additional extractions using the same procedure. The combined filtrates collected from all three extractions were filtered and concentrated by rotary evaporation at 40 °C. The resulting concentrate was then freeze-dried using the relevant equipment.

The crude extract was dissolved in an appropriate solvent, filtered, and purified using an XAD-1600 macroporous resin column. A certain amount of the crude extract was loaded into the column and allowed to absorb for 1 hour to ensure complete absorption. Unabsorbed impurities were washed away with 5L of distilled water, followed by elution with a 70% ethanol solution. Once the eluant became colorless, it was collected, concentrated, and freeze-dried to obtain the LBTE.

Molecular docking analysis

Molecular docking analysis was performed to assess the interactions between core compounds and the crystal structures of key proteins in this study. The preparation of target protein was conducted using Molecular Operating Environment (MOE) software, following standard procedure with default parameters. This included the removal of all the water molecules and the identification of binding active site by specifying the atoms of a native ligand. The “induced fit” protocol was employed, which allows the side chains of the receptor’s binding site to adjust according to ligand conformations, while a positional constraint was applied (weight: 10). Firstly, all docked poses were ranked using the London dG scoring function. Subsequently, the top 30 poses underwent force field refinement, followed by rescoring with the GBVI/WSA dG scoring function. The conformation with the lowest S score was identified as the most probable binding mode. In MOE software, a lower S score corresponds to higher binding affinity between protein receptors and ligand compounds.

Animal model and treatment

Twelve male KM mice (18-22 g) were purchased from SPF (Beijing)

Biotechnology Co., Ltd. (license No.110324230101502642). The study protocols were approved by the Animal Experimental Ethics Committee of Guangxi University (No. GXU-2023-0226), and all animal procedures in the study were performed in accordance with the local guidelines and regulations.

After one week of adaptive feeding, the male mice were randomly assigned to two groups to establish two different models: high-fat diet (HFD) + streptozotocin (STZ) (50 mg/kg) and HFD + STZ (100 mg/kg). The mice were fed with an HFD for four weeks. Subsequently, the mice fed with an HFD received an intraperitoneal injection of STZ at a dose of 50 mg/kg and 100mg/kg, respectively. Following the injection, fasting blood glucose (FBG) levels were measured using a glucometer, and mice with FBG levels ≥ 11.1 mmol/L were considered as having type 2 diabetes (T2D).

Subsequently, both groups were treated with metformin at a dose of 200 mg/kg of metformin via intragastric administration. FBG was measured weekly using the tail-bleed method in all mice following overnight fasting throughout the two-week treatment period.

In addition, the effects of two different metformin administration methods on FBG levels were observed. The first method involved a single administration of metformin, with FBG levels measured at 0, 3, 4 hours post-administration to observe the immediate trends. The second method involved administering metformin three times at three-hour intervals, with FBG levels similarly measured at 0, 3, 4 hours post-administration.

Gut microbiota analysis

The V3 - V4 regions of the bacterial 16S rRNA genes were amplified with universal primers 343F (5’-TACGGRAGGCAGCAG-3’) and 798R (5’-AGGGTATCTAATCCT-3’). The PCR-amplified library was purified using Agencourt AMPure XP beads and amplified for another round of PCR. After purification, the final amplicon was quantified using the Qubit dsDNA assay kit (Life Technologies, Q32854). The purified amplicon was pooled in equal amounts for sequencing. 16S rRNA gene sequencing of the fecal DNA was performed on an Illumina MiSeq platform (MiSeq PE300, Illumina, USA).

Post-sequencing, clean sequences were obtained after filtering and chimera removal. Representative read of each Amplicon Sequence Variants (ASV) was selected using the QIIME2 package. α-diversity and β-diversity indices were calculated within QIIME2 software to evaluate microbial community structure based on these analyses.

The preparation and GC-MS analysis of short-chain fatty acids (SCFAs)

SCFA extraction: 90 mg of fecal samples were mixed with 300 μL OmniSolv pure water and vortex for 20 seconds, repeating the process three times using Vortex Mixer. The mixture was then incubated at 4 °C with shaking for 30 minutes, followed by centrifugation at 13,000×g for 30 minutes. Subsequently, 200 μL of supernatant (fecal homogenate) was transferred into a new microtube containing 10 μL of 5 M HCl to adjust the pH to 2. The acidified fecal samples were extracted by adding 100 μL of anhydrous diethyl ether (DE; 2:1, v/v), vortexed, and incubated on ice for 5 minutes, and then centrifuged at 10,000×g for 5 minutes. The DE layer (containing SCFAs) was transferred to a new microtube containing anhydrous Na_2SO_4 to remove the residual water. The remaining aqueous layer was further extracted with DE for two additional times. The DE layers were pooled and mixed for further derivatization.

Derivatization procedure: A 100 μL aliquot of DE extract was accurately transferred into a glass insert in a GC vial, followed by the addition of 5 μL of BSTFA. The mixture was incubated at 37 °C for 2 hours in the GC vial. Then, derivatized samples were loaded onto the GC-MS analysis. Pure water was used as a blank sample to correct the background, processed in the same manner as the fecal samples.

GC-MS analysis: the analysis was conducted using an 7250 gas chromatograph coupled with an 8890 mass selective detector (Agilent Technologies, Santa Clara, CA, USA) equipped with an HP-5 ms capillary column (30 m × 0.25 mm × 0.25 μm film thickness). The temperature of injector, ion source, quadrupole, and the GC-MS interface were set to 260, 230, 150, and 280 °C, respectively. The flow rate of helium carrier gas was maintained at 1 ml/min. A 1 μL aliquot of the derivatized sample was injected with a solvent delay time of 3 minutes with a split ratio of 10:1. The initial column temperature was set at 40 °C, held for 2 minutes, ramped to 150 °C at a rate of 15 °C/min, held for 1 minute, and then finally increased to 280 °C at a rate of 30 °C/min, where it was held for 5 minutes. Analytes were ionized in the electron impact (EI) mode at 70 eV.

Supplementary Figures and Tables

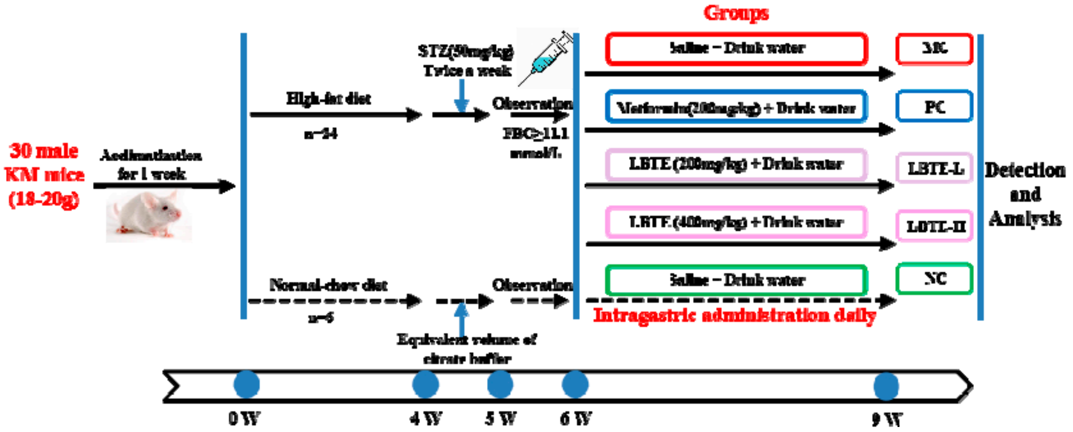

Figure S1. Schematic diagram of the experimental design.

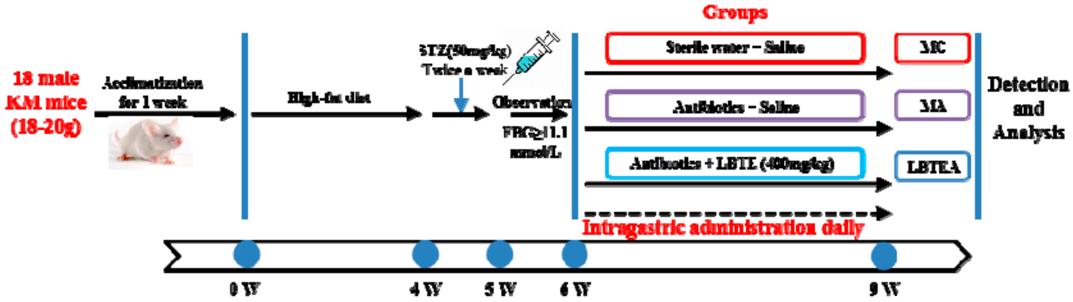

Figure S2. Schematic diagram of the experimental design for pseudo-germ-free mouse.

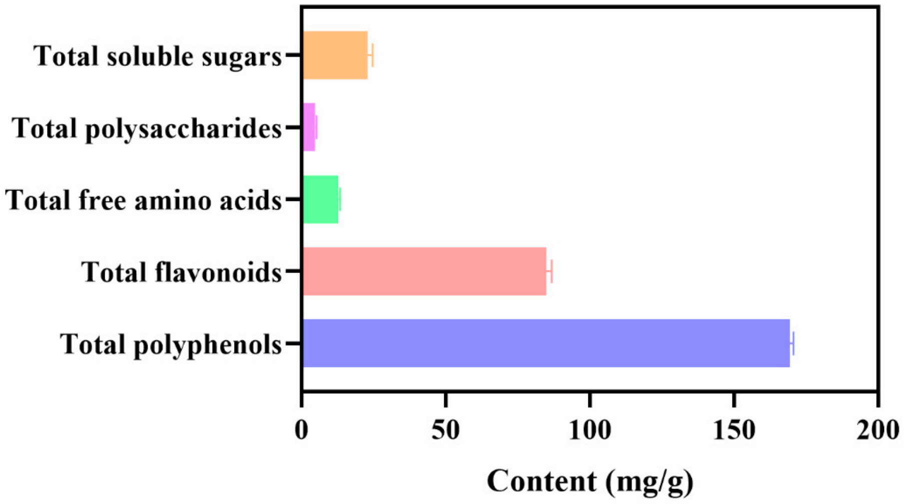

Figure S3. Primary chemical constituents of LBTE. LBTE, Liubao tea extract.

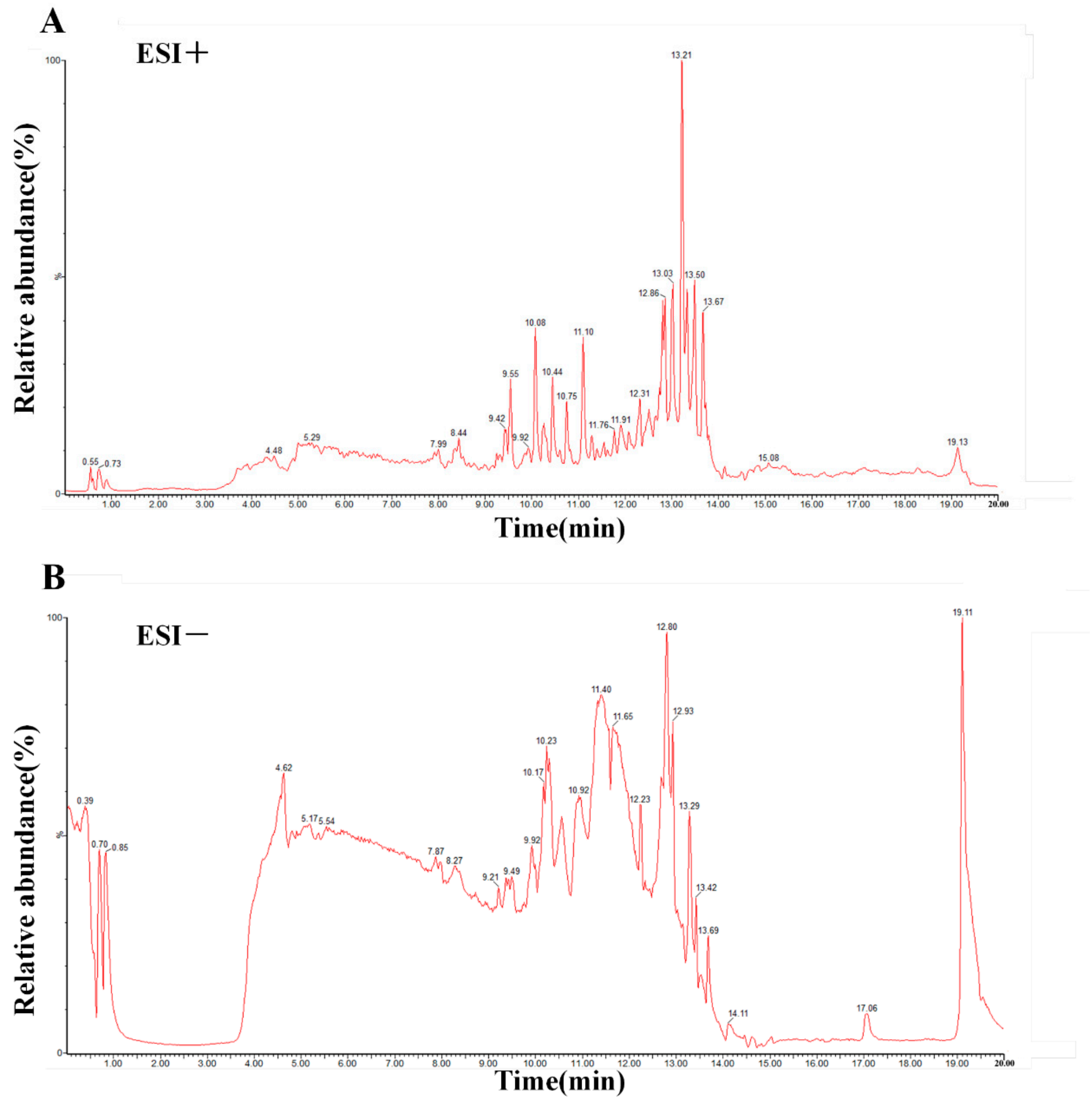

Figure S4. Total ion chromatogram (TIC) of compounds in LBTE detected by LC-MS (**A, B**). TIC in the ESI+ and ESI- modes, respectively.

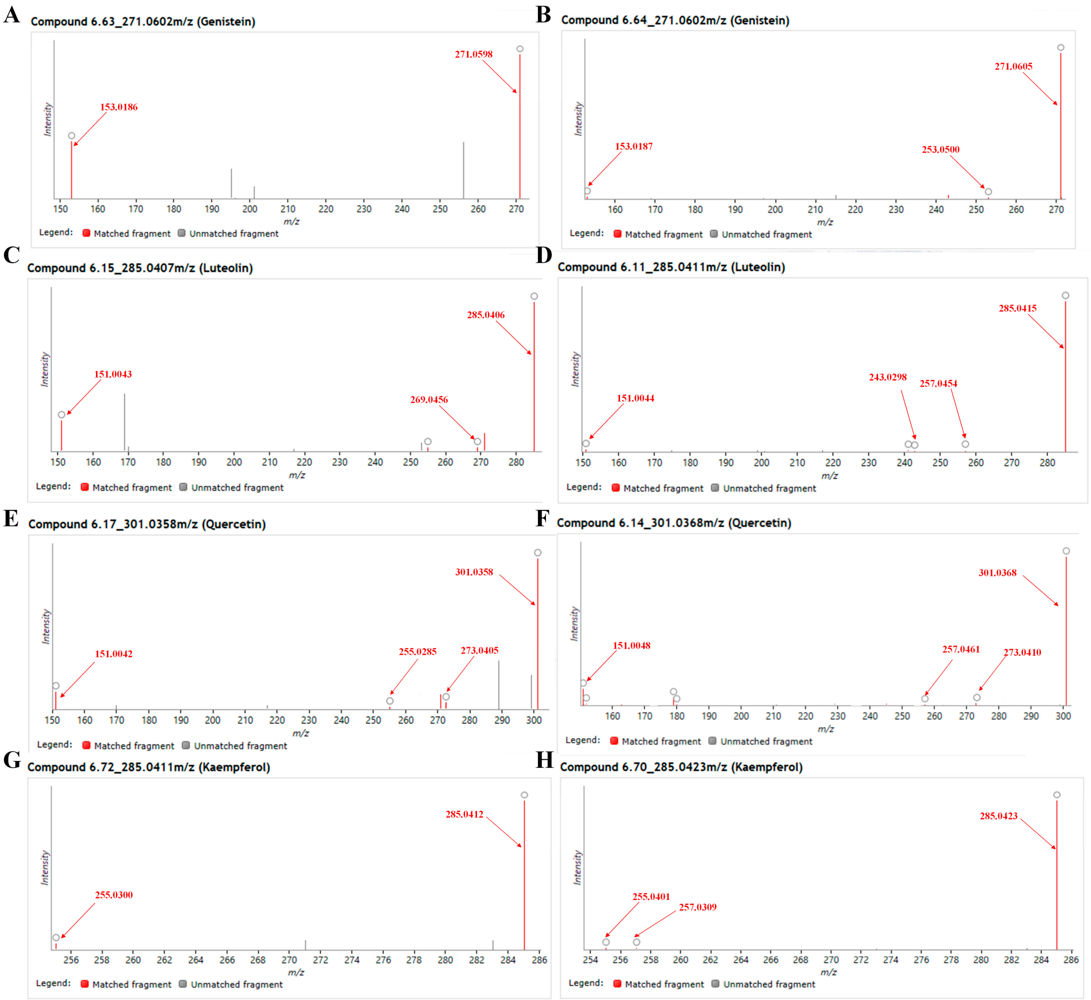

Figure S5. Identification of key active ingredients in LBTE. (**A**) Fragment ion diagram of genistein in the sample. (**B**) Fragment ion diagram of standard genistein. (**C**) Fragment ion diagram of luteolin in the sample. (**D**) Fragment ion diagram of standard luteolin. (**E**) Fragment ion diagram of quercetin in the sample. (**F**) Fragment ion diagram of standard quercetin. (**G**) Fragment ion diagram of kaempferol in the sample. (**H**) Fragment ion diagram of standard kaempferol.

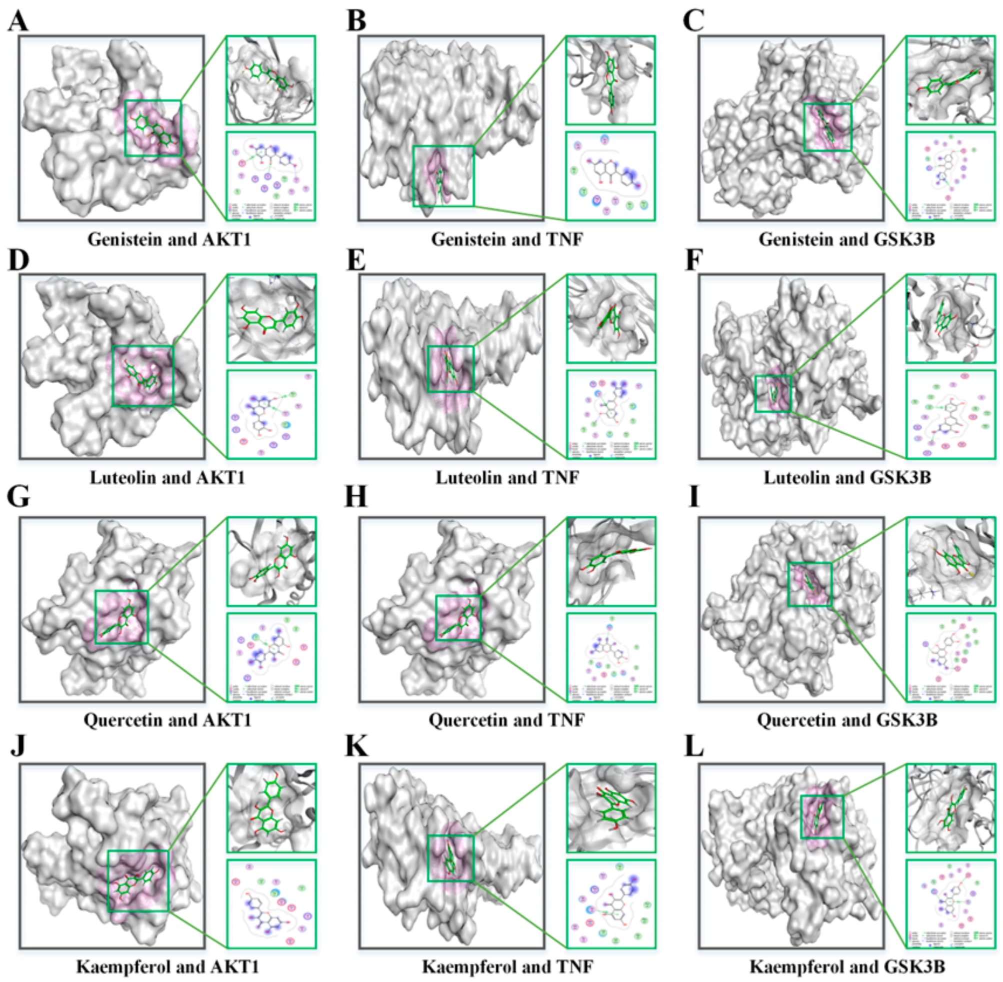

Figure S6. Molecular docking analysis of the key targets and key core compounds. (**A**) Genistein-AKT1, (**B**) Genistein-TNF, (**C**) Genistein-GSK3B, (**D**) Luteolin-AKT1, (**E**) Luteolin-TNF, (**F**) Luteolin-GSK3B, (**G**) Quercetin-AKT1, (**H**) Quercetin-TNF, (**I**) Quercetin-GSK3B, (**J**) Kaempferol-AKT1, (**K**) Kaempferol-TNF, (**L**) Kaempferol-GSK3B.

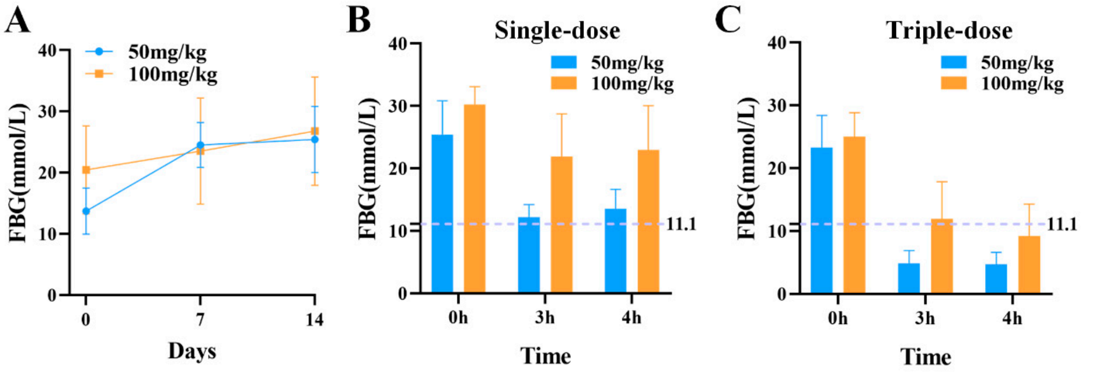

Figure S7. Effects of different modes of metformin administration on blood glucose in mice. (**A**) Changes in blood glucose with different methods of modeling. (**B**) Fasting blood glucose after the administration of single-dose of metformin (200mg/kg). (**C**) Fasting blood glucose after the administration of triple-dose of metformin (200mg/kg) at 3h intervals. 50 mg/kg, high-fat diet + STZ (50 mg/kg); 100 mg/kg, high-fat diet + STZ (100 mg/kg). Data are expressed as mean ± standard deviation (SD) (n = 5).

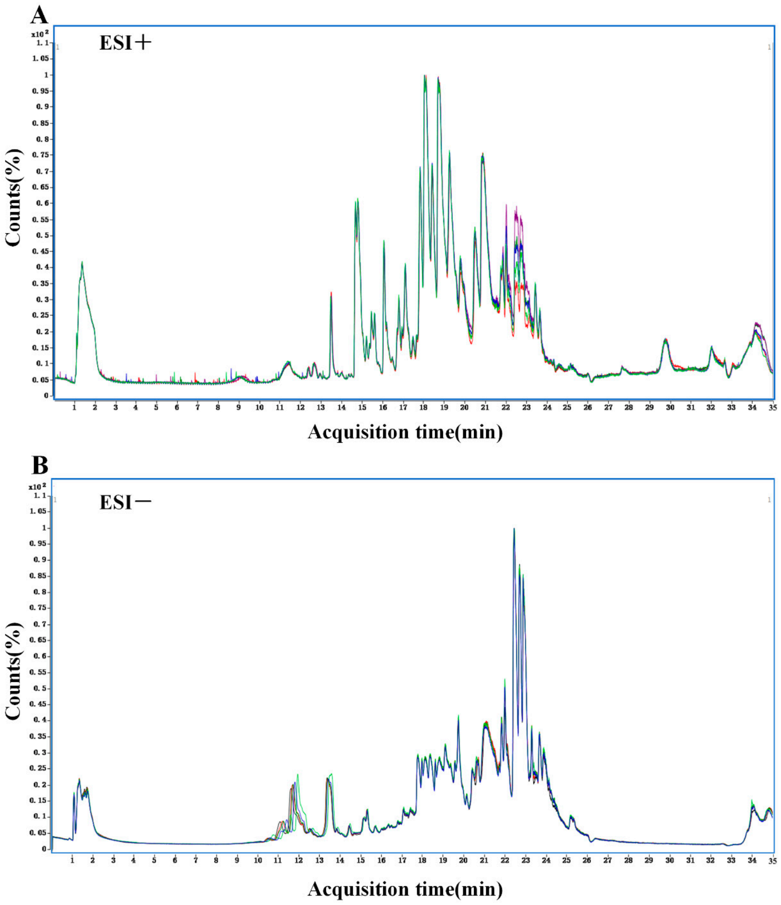

Figure S8. Reproducibility assessment of QC samples in the serum metabolomic analysis. (**A, B**) TIC of QC samples in the ESI+ and ESI- modes, respectively.

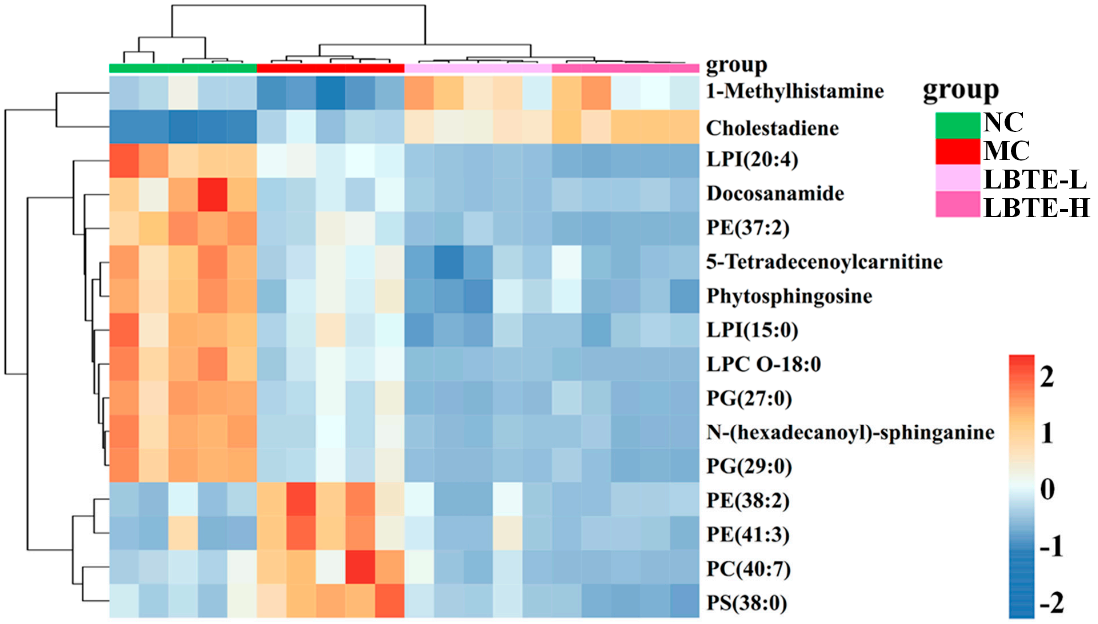

Figure S9. Level distribution of serum differential metabolites shared in the comparison among the four groups. Red and blue colors represent relative increase and decrease values, respectively.

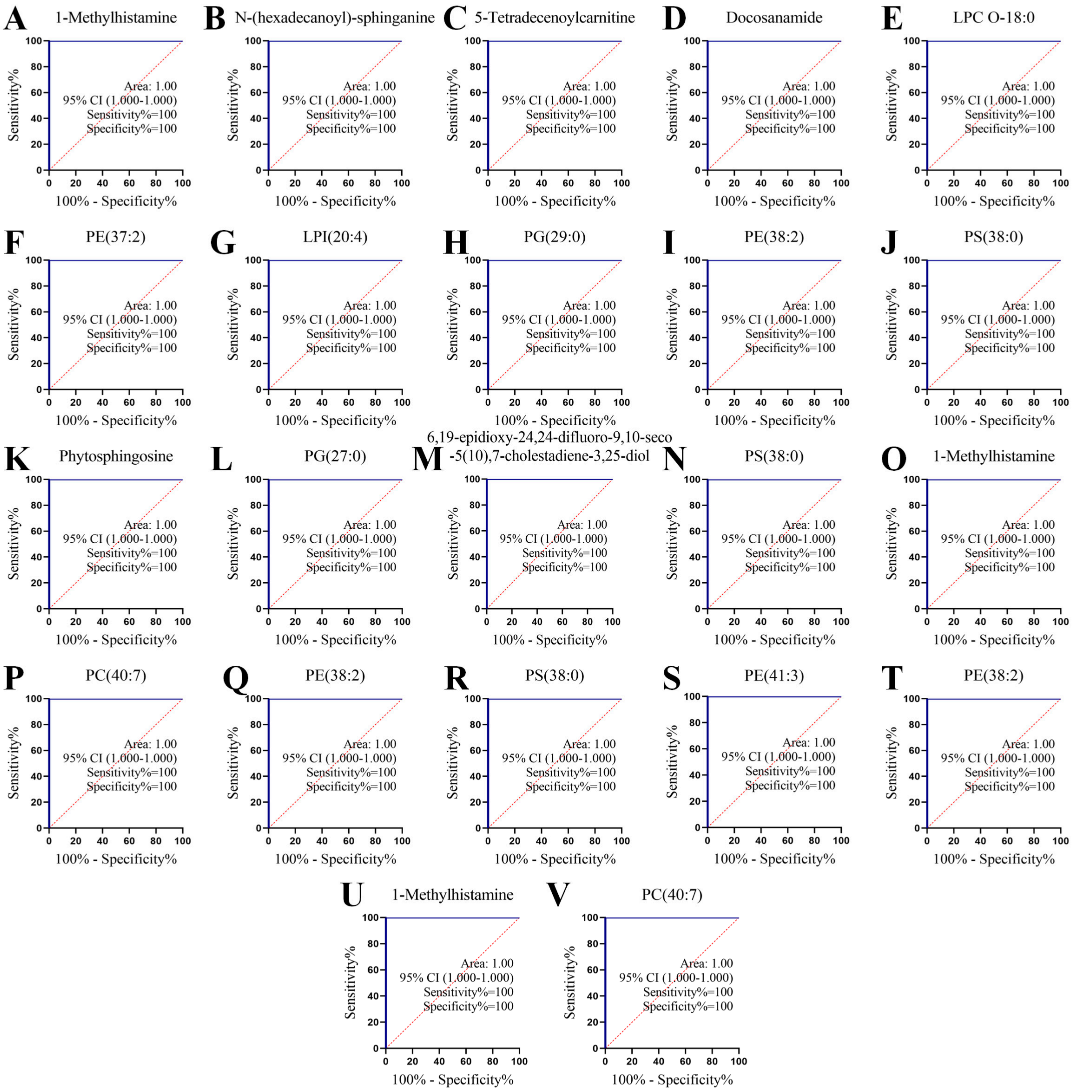

Figure S10. ROC analysis to evaluate the discriminative performance of serum differential metabolites between two groups. The selected criteria were set as AUC > 0.950 and FC > 5.00. Performance evaluation of serum differential metabolites between NC and MC groups (**A–M**), LBTE-L and MC groups (**N–Q**), LBTE-H and MC groups (**R–V**), respectively.

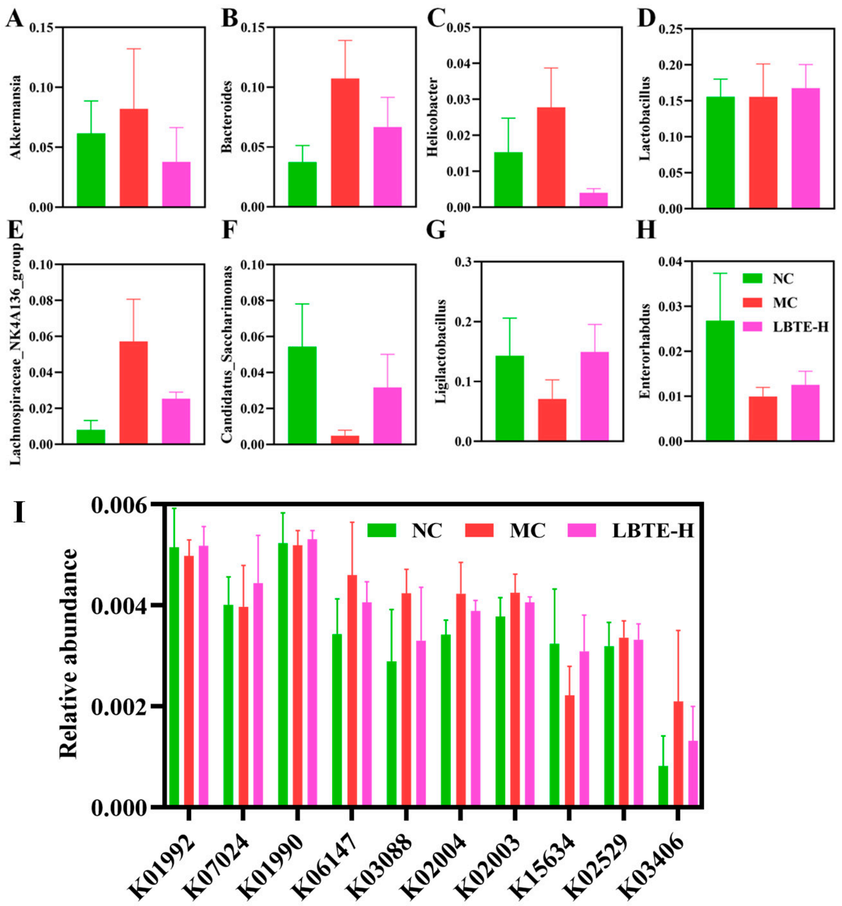

Figure S11. Analysis of discriminative gut microbiota and KEGG functional enrichment. (**A–H**) Relative abundance differences of gut microbiota at the genus level. (**I**) KEGG functional enrichment relative abundance differences based on PICRUSt2 results. K01992: ABC-2.P; ABC-2 type transport system permease protein. K07024: SPP, sucrose-6-phosphatase [EC:3.1.3.24]. K01990: ABC-2.A, ABC-2 type transport system ATP-binding protein. K06147: ABCB-BAC, ATP-binding cassette, subfamily B. K03088: rpoE, RNA polymerase sigma-70 factor, ECF subfamily. K02004: ABC.CD.P, putative ABC transport system permease protein. K02004: ABC.CD.P, putative ABC transport system permease protein. K02003: ABC.CD.A, putative ABC transport system ATP-binding protein. K15634: gpmB, probable phosphoglycerate mutase [EC:5.4.2.12]. K02529: lacI; LacI family transcriptional regulator. K03406: mcp, methyl-accepting chemotaxis protein. Data are presented as mean ± SD (n = 5).

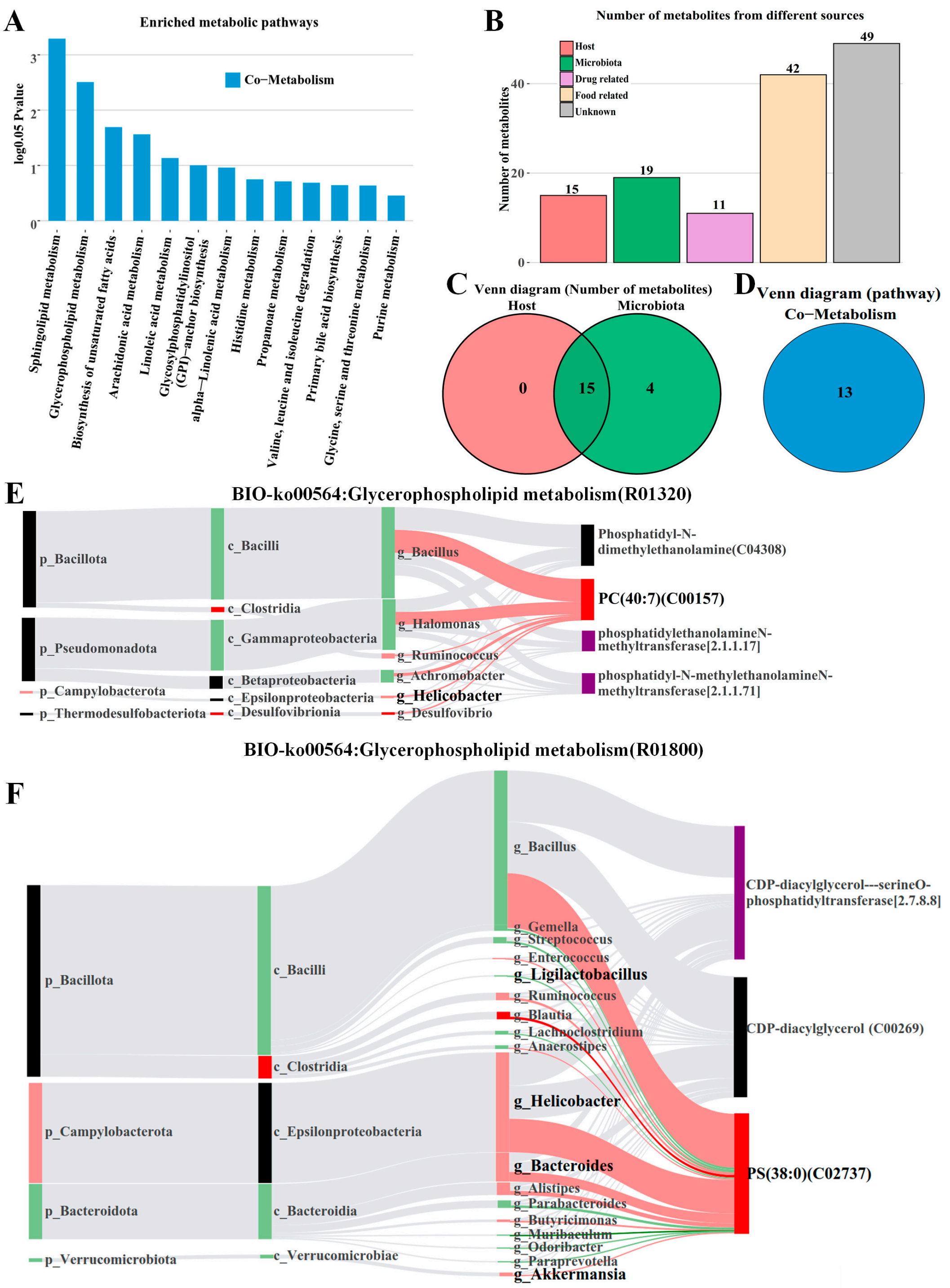

Figure S12. MetOrigin analysis on gut microbiota and differential metabolites between NC and MC groups. (**A,D**) Number of enriched metabolic pathways. (**B,C**) Number distribution of metabolites in different categories. (**E,F**) The Bio-Sankey Network for R01320 and R01800 metabolic reaction in glycerophospholipid metabolism. Red and green color of nodes indicate upregulation and downregulation, respectively. Red and green bands indicate positive and negative correlations with metabolites, respectively. Dark red or green colors indicate significant difference, $P < 0.05$.

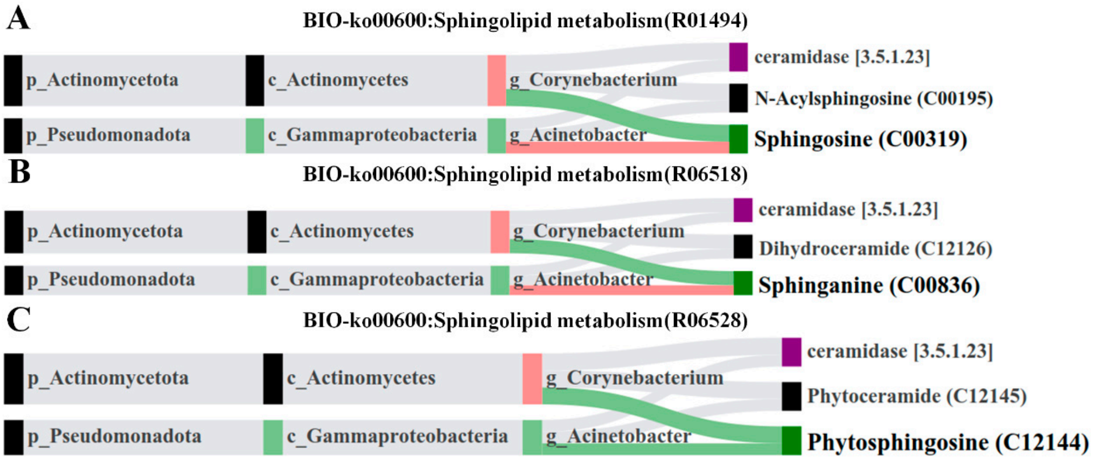

Figure S13. BIO-Sankey Network of gut microbiota and differential metabolites between the NC and MC groups. (**A–C**) The Bio-Sankey Network for R01494, R06518 and R06528 metabolic reaction in sphingolipid metabolism. Red and green color of nodes indicate upregulation and downregulation, respectively. Red and green bands indicate positive and negative correlations with metabolites, respectively. Dark red or green colors indicate significant difference, $P < 0.05$.

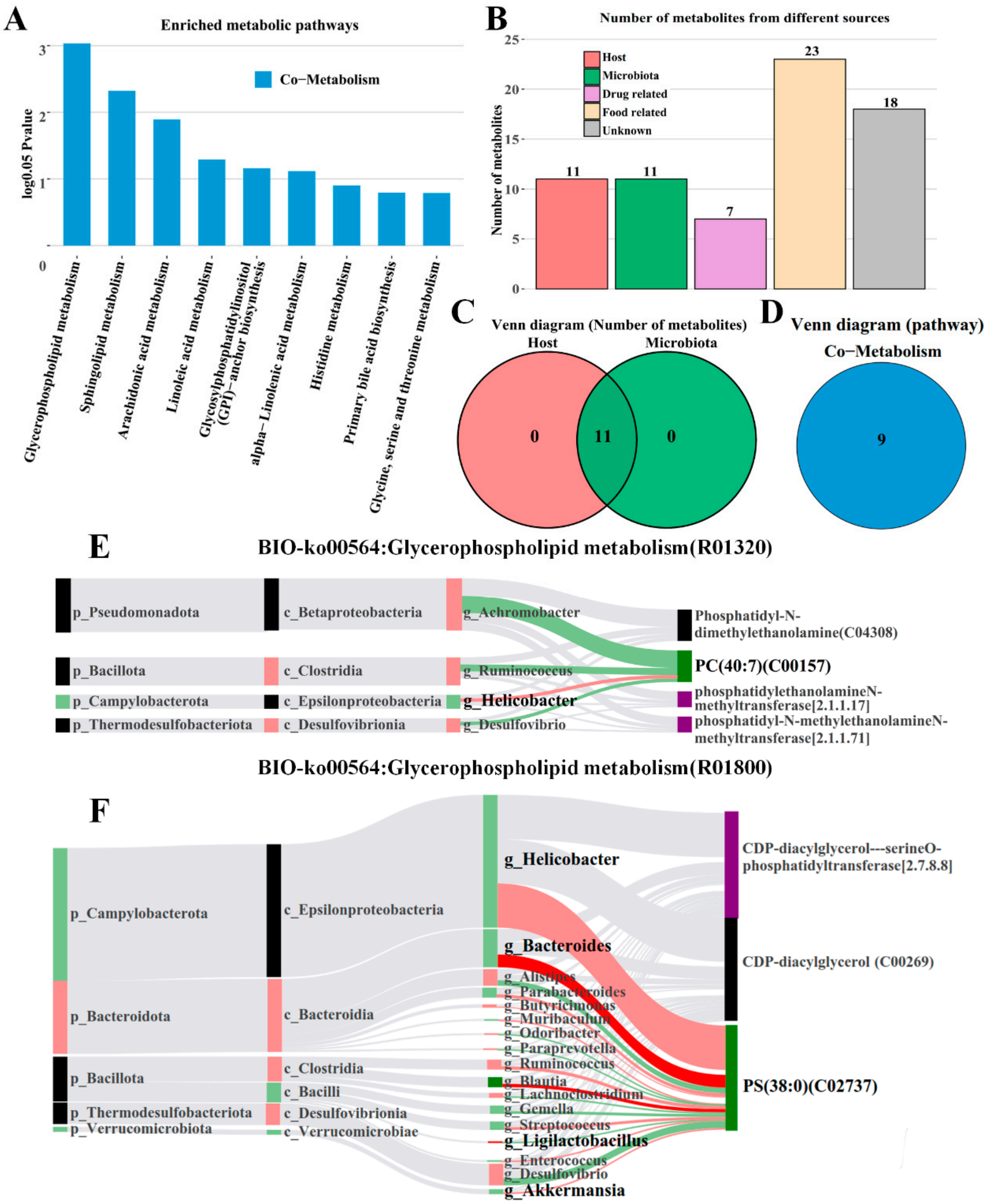

Figure S14. MetOrigin analysis on gut microbiota and differential metabolites between MC and LBTE-L groups. (**A,D**) Number of enriched metabolic pathways. (**B,C**) Number distribution of metabolites in different categories. (**E,F**) The Bio-Sankey Network for R01320 and R01800 metabolic reaction in glycerophospholipid metabolism. Red and green color of nodes indicate upregulation and downregulation, respectively. Red and green bands indicate positive and negative correlations with metabolites, respectively. Dark red or green colors indicate significant difference, $P < 0.05$.

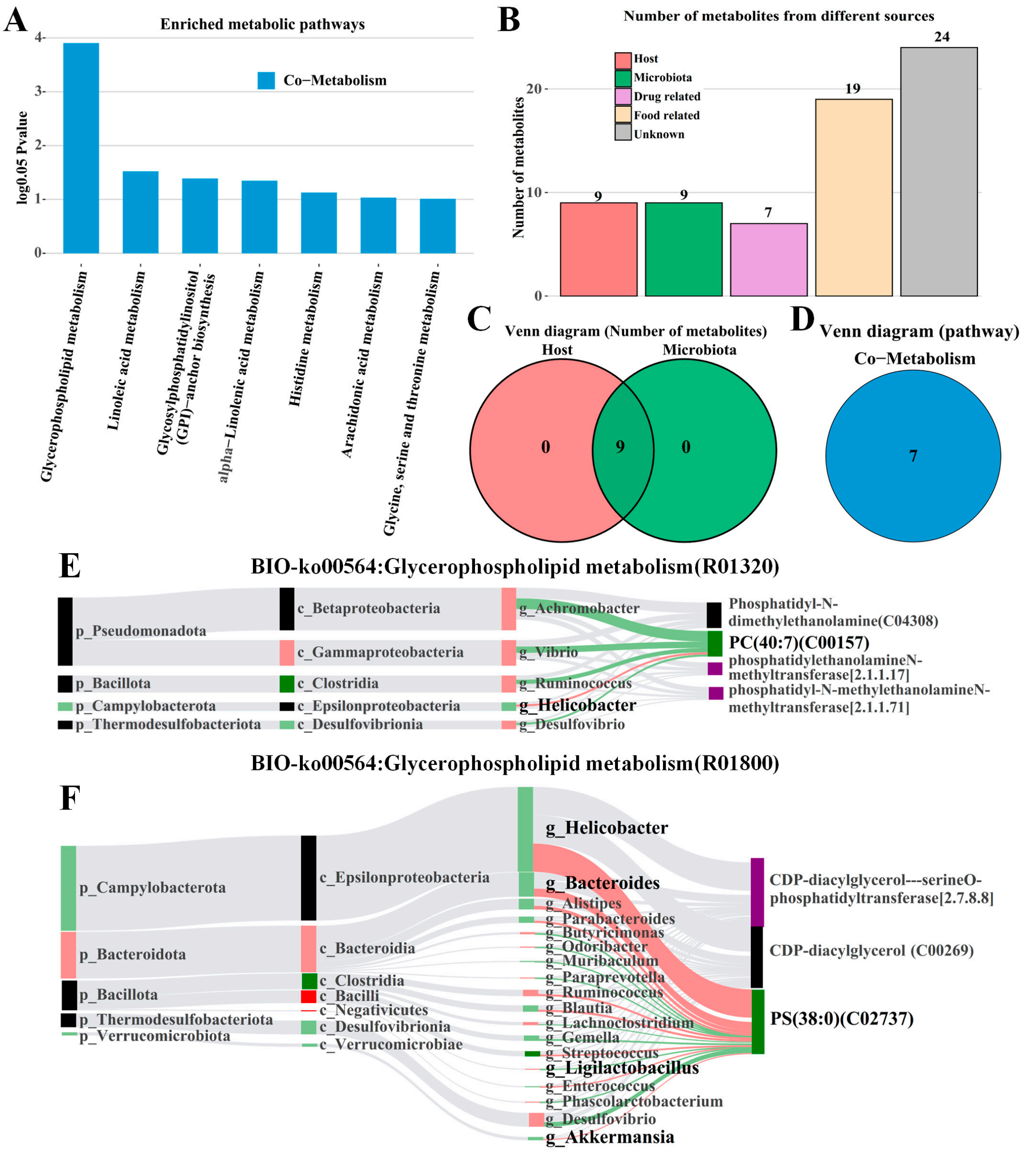

Figure S15. MetOrigin analysis on gut microbiota and differential metabolites between MC and LBTE-H groups. (**A,D**) Number of enriched metabolic pathways. (**B,C**) Number distribution of metabolites in different categories. (**E,F**) The BIO-Sankey Network for R01320 and R01800 metabolic reaction in glycerophospholipid metabolism. Red and green color of nodes indicate upregulation and downregulation, respectively. Red and green bands indicate positive and negative correlations with metabolites, respectively. Dark red or green colors indicate significant difference, $P < 0.05$.

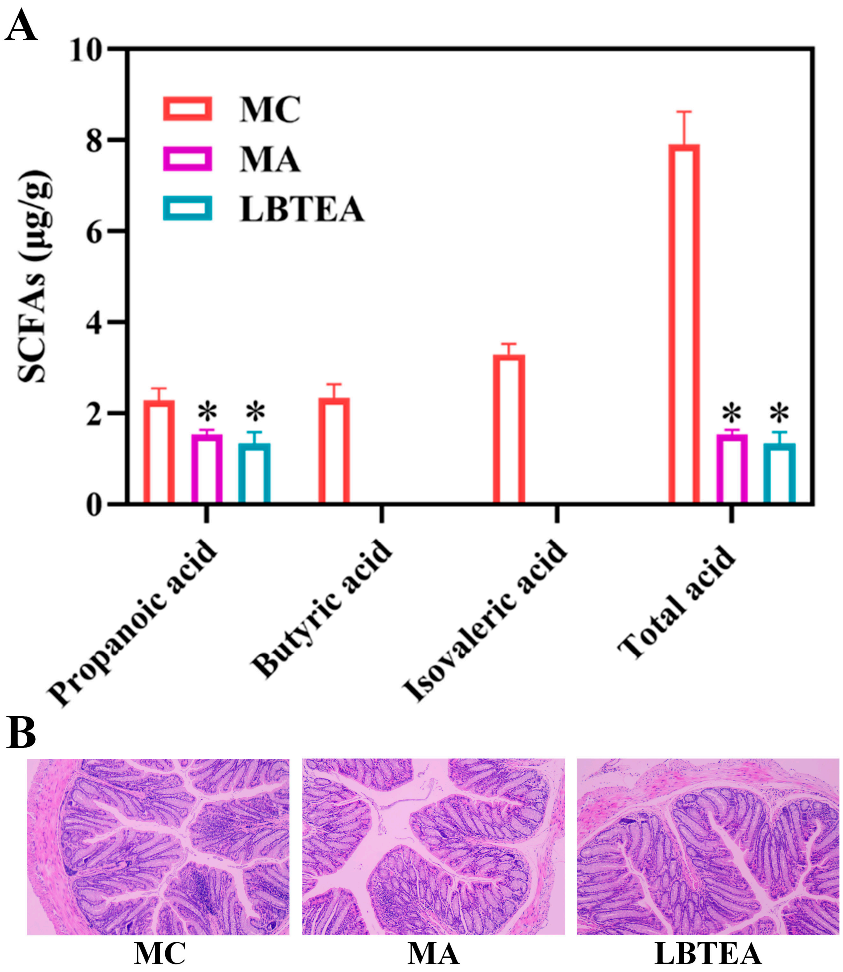

Figure S16. Effects of LBTE treatment on the short-chain fatty acids (SCFAs) levels and the intestinal barrier function. (**A**) The levels of SCFAs. (**B**) H&E staining of colon tissue (100 ×). MC, high-fat diet (HFD); MA, HFD + antibiotic; LBTEA, HFD + antibiotic + LBTE (400 mg/kg). Data are expressed as mean ± standard deviation (SD) (n = 5). * $P < 0.05$ compared with the MC group.

Table S1. Chromatographic conditions for chemical composition analysis of LBTE.

Time (min)	Flow rate (mL/min)	Mobile phase A (%)	Mobile phase B (%)	Injection volume (μL)
0	0.4	98	2	5
2	0.4	98	2	
13	0.4	0	100	
18	0.4	0	100	
20	0.4	98	2	

Analytical conditions: Waters Acquity UPLC HSS T3 (1.8 μm, 2.1 mm × 100 mm) at 40 °C column temperature. Mobile phases: A was ultrapure water and B was acetonitrile, both of which contained 0.1% formic acid in ESI+ and ESI- modes.

Table S2. Mass spectrometric conditions for chemical composition analysis of LBTE.

Parameters	Values
Capillary voltage	2000 V
Cone	40 V
Source temperature	150 °C
Desolvation temperature	500 °C
Cone gas	50 L/h
Desolvation gas	1000 L/h
Nebuliser gas	6 bar
Scan range	100-1200 *m/z*

Table S3. Chromatographic conditions for quantitative analysis of potential ingredients in LBTE.

Time (min)	Flow rate (mL/min)	Mobile phase A (%)	Mobile phase B (%)	Injection volume (μL)
0	0.4	68	32	10
20	0.4	60	40	
24	0.4	68	32	
27	0.4	68	32	

Analytical conditions: Agilent Eclipse Plus C18 (3.0 × 100 mm) at 30 °C column temperature. Mobile phases: A was ultrapure water with 0.1% formic acid and B was acetonitrile in ESI+ and ESI- modes.

Table S4. Mass spectrometric conditions for quantitative analysis of potential ingredients in LBTE.

Parameters	Values
Capillary voltage	3500 V
Fragmentor voltage	100 V
Sheath gas flow	11 L/min
Gas flow	8 L/min
Gas temperature	300 °C
Sheath gas temperature	350 °C
Nebulizer pressure	40 psi
Scan mode	Full scan
Full scan range	100-1100 *m/z*

Table S5. Chromatographic conditions for serum metabolomic analysis.

Time (min)	Flow rate (mL/min)	Mobile phase A (%)	Mobile phase B (%)	Injection volume (μL)
0	0.4	90	10	5
4	0.4	90	10	
20	0.4	0	100	
30	0.4	0	100	
35	0.4	90	10	

Analytical conditions: Waters Acquity UPLC BEH C18 (1.7 μm, 2.1 mm × 100 mm) at 40 °C column temperature. Mobile phases: A was ultrapure water and B was acetonitrile, both of which contained 0.1% formic acid in ESI+ mode and 0.01% formic acid in ESI- mode.

Table S6. Mass spectrometric conditions for serum metabolomic analysis.

Parameters	Values
Capillary voltage	3500 V
Fragmentor voltage	140 V
Sheath gas flow	11 L/min
Gas flow	8 L/min
Gas temperature	300 °C
Sheath gas temperature	300 °C
Nebulizer pressure	35 psi
Scan mode	Full scan and Auto MS/MS scan
Full scan range	100-1500 *m/z*
Auto MS/MS scan range	50-1500 *m/z*

Table S7. Primer sequences for the qPCR analysis.

Gene	Forward Primer (5′-3′)	Reverse Primer (5′-3′)
GAPDH	CATCACTGCCACCCAGAAGACTG	ATGCCAGTGAGCTTCCCGTTCAG
IL-6	GGACTGATGCTGGTGACAACC	TGTTGGGAGTGGTATCCTCTGT
IL-10	AAGGGTTACTTGGGTTGCC	CACAGGGGAGAAATCGTGACAACC
AKT1	CAAGAAGGAGGTCATCGTCGC	AGTCCAGGGCAGACACAATCT
GSK3B	GACCGAGAACCACCTCCTTTG	TGCTGCCATCTTTATCTCTGCT
TNF-α	CCAGACCCTCACACTCACAA	TGTCCCTTGAAGAACCTG
AGPAT	CATGCTGCCCTTCAAACGAG	CTGACAACGTCCAGGCGAG
GPAT	ACAGTTGGCACAATAGACGTTT	CCTTCCATTTCAGTGTTGCAGA
PAP	GAATTGCCACAGGCTGCAAAG	CATTGTTGGCGACTGGTCACT
GPR41	TTCCTCCAAGTTCCAAGCCG	GGCTGACTTGCTGAGTCCAA
GPR109A	TCCAAGTCTCCAAAGGTGGT	TGTTTCTCTCCAGCACTGAGTT
ZO-1	TTGAAAGTCCACCTCCTTACAGA	CCGGATAAAAAGAGTACGCTGG
Occludin	TGTTTATGCGGACGGTGGC	GCTGTTTCCTCCATTGCTGTG

Table S8. Characterization of compounds in LBTE via LC-MS.

NO.	Identification	Ion mode	RT (min)	Adduct	Observed mass	Error (ppm)	Formula	Fragment ions
1	Tricetin	+	6.17	M+H	303.0497	0.08	$C_{15}H_{10}O_7$	275.0548、287.0555、303.0498
2	Quercetin	-	6.17	M-H	301.0358	1.51	$C_{15}H_{10}O_7$	151.0042、255.0285、301.0358
3	Kaempferol	-	6.72	M-H	285.0412	2.15	$C_{15}H_{10}O_6$	255.0300、285.0412
4	Luteolin	-	6.15	M-H	285.0406	0.95	$C_{15}H_{10}O_6$	151.0043、269.0456、285.0406
5	Norartocarpetin	+	5.29	M+H	287.0554	0.55	$C_{15}H_{10}O_6$	258.0518、287.0554
6	Apigenin	+	6.63	M+H	271.0608	1.11	$C_{15}H_{10}O_5$	153.0183、271.0608
7	3,4',7-Trihydroxyflavone	+	4.74	M+H	271.0606	0.69	$C_{15}H_{10}O_5$	163.0395、255.0658、271.0606
8	Chrysin	+	4.62	M+H	255.0670	6.83	$C_{15}H_{10}O_4$	237.0564、255.0670
9	Genistein	+	6.63	M+H	271.0598	0.51	$C_{15}H_{10}O_5$	153.0186、271.0598
10	3'-Hydroxygenistein	+	5.29	M+H	287.0554	0.55	$C_{15}H_{10}O_6$	258.0518、287.0554
11	Pinocembrin	+	5.89	M+H	257.0810	0.54	$C_{15}H_{12}O_4$	163.0386、213.0548
12	Naringenin	+	4.79	M+H	273.0762	1.37	$C_{15}H_{12}O_5$	153.0190、258.0486、273.0762
13	5-Hydroxyflavone	+	5.38	M+Na	423.1075	7.18	$C_{21}H_{20}O_8$	205.0499、339.0866、423.1075
14	3'-Hydroxydaidzein	-	7.16	M+FA-H	315.0523	1.83	$C_{15}H_{10}O_5$	285.0407、315.0523
15	2'-Hydroxygenistein	-	4.55	M+FA-H	315.0514	2.49	$C_{15}H_{10}O_6$	138.0342、163.0040、315.0514
16	Caffeine	+	4.70	M+H	195.0877	0.07	$C_8H_{10}N_4O_2$	195.0878、233.0447
17	Isorhamnetin	-	5.32	M-H_2O-H	297.0386	5.79	$C_{16}H_{12}O_7$	284.0327、287.0563、297.0386
18	Epigallocatechin	+	4.94	M+H	329.0658	2.63	$C_{15}H_{14}O_7$	307.0804、329.0658
19	Chrysoeriol	+	5.07	M+H	301.0707	0.16	$C_{16}H_{12}O_6$	259.0596、285.0746、301.0707
20	Diosmetin	+	4.50	M+H	301.0706	0.51	$C_{16}H_{12}O_6$	163.0391、271.0598、301.0706
21	Catechin	+	4.45	M+H	291.0861	0.88	$C_{15}H_{14}O_6$	291.0861、313.0797
22	Nepetin	-	4.24	M+FA-H	361.0580	4.70	$C_{16}H_{12}O_7$	193.0151、305.0669、333.0617
23	Sinensetin	-	4.85	M+FA-H	433.1143	0.79	$C_{20}H_{20}O_8$	163.0391、247.0601、287.0547
24	Epicatechin	+	5.49	M+H	291.0861	0.61	$C_{15}H_{14}O_6$	291.0861、313.0678
25	Daidzein	+	6.42	M+H	255.0666	5.53	$C_{15}H_{10}O_4$	210.0677、239.0712、255.0666
26	7-Hydroxy-3',4',5,6-tetramethoxyflavone	-	6.32	M-H	357.0989	5.45	$C_{19}H_{18}O_7$	177.0202、205.0518、357.0989
27	Luteolin 7-methyl ether	+	7.40	M+H	301.0710	0.15	$C_{16}H_{12}O_6$	289.0699、301.0710

28	Acerosin	+	6.05	M+H-H_2O	343.0820	2.42	$C_{18}H_{16}O_8$	191.0343、303.0868、343.0820
29	Eriodictyol	-	4.76	M+FA-H	333.0621	2.29	$C_{15}H_{12}O_6$	179.0357、287.0563、333.0621
30	Eupatilin	+	5.38	M+Na	367.0814	7.35	$C_{18}H_{16}O_7$	189.0548、297.0749、343.0809
31	Limocitrol	-	3.69	M-H	375.0699	3.34	$C_{18}H_{16}O_9$	327.0493、347.0777、375.0699
32	Tangeritin	+	6.14	M+Na	395.1121	5.39	$C_{20}H_{20}O_7$	147.0439、26.0760、355.1173
33	Dihydrogenistein	+	5.41	M+H	273.0759	1.52	$C_{15}H_{12}O_5$	167.0335、257.0453、273.0759
34	Pedalitin	-	4.15	M+FA-H	361.0573	2.45	$C_{16}H_{12}O_7$	177.0202、287.0562、349.0587

Table S9. Identification information of the potential bioactive ingredients in LBTE against T2D.

Compound name	Formula	Source	Score	RT (min)	Ion mode	Adduct	Error (ppm)	Fragment ions
Genistein	$C_{15}H_{10}O_5$	Reference standard	45.1	6.64	+	M+H	1.14	149.0235、153.0187、253.0500、271.0605
		LBTE	41.7	6.63	+	M+H	0.51	153.0186、271.0598
Luteolin	$C_{15}H_{10}O_6$	Reference standard	49.9	6.15	-	M-H	3.58	151.0046、255.0306、257.0454、285.0415
		LBTE	49.7	6.15	-	M-H	0.95	151.0043、269.0456、285.0406
Quercetin	$C_{15}H_{10}O_7$	Reference standard	53.1	6.14	-	M-H	1.41	151.0048、257.0461、273.0410、301.0369
		LBTE	43.4	6.17	-	M-H	1.51	151.0042、255.0285、301.0358
Kaempferol	$C_{15}H_{10}O_6$	Reference standard	48.3	6.70	-	M-H	6.39	151.0047、211.0411、255.0309、285.0423
		LBTE	42.3	6.72	-	M-H	2.15	255.0300、285.0412

Table S10. Interaction between the key proteins and the potential bioactive compounds from LBTE.

**Compound**	**AKT1(1UNQ)**			**TNF(5UUI)**			**GSK3B(1O6L)**		
	S score	H-Bonds	π-Interactions	S score	H-Bonds	π-Interactions	S score	H-Bonds	π-Interactions
**Quercetin**	-4.316	--	H-pi	-4.516	H-acceptor	--	-4.982	H-donor<br>H-donor<br>H-acceptor	--
**Kaempferol**	-4.491	--	--	-4.521	--	pi-H	-4.913	H-donor<br>H-acceptor	pi-H
**Luteolin**	-4.360	--	H-pi<br>pi-H	-4.457	H-donor	pi-H	-5.290	H-donor	pi-H
**Genistein**	-4.264	H-acceptor<br>H-acceptor	pi-H	-4.592	--	--	-4.907	H-donor<br>H-acceptor	pi-H
